# Confirmation and reproducibility of endotracheal tube position by tracheal ultrasonography in preterms: expert versus trainee

**DOI:** 10.1007/s00431-025-06626-3

**Published:** 2025-12-09

**Authors:** Mariam John Amin Ibrahim, Mohamed Nasr El Dine Elbarbary, George Ezzat Elkess Yacoub, Mona Abdo Mostafa Abdo

**Affiliations:** 1 Pediatrics Department, Faculty of Medicine, Ain Shams University, Cairo, Egypt; 2 Diagnostic Radiology Department, Faculty of Medicine, Ain Shams University, Cairo, Egypt; 3 Neonatology Resident in Ministry of Health and Population, Cairo, Egypt

**Keywords:** Tracheal ultrasonography, Tracheal intubation, Preterm neonate, X-ray chest

## Abstract

Tracheal intubation (TI) is critical in neonatal intensive care and delivery rooms, requiring precise placement of the endotracheal tube (ETT) to prevent complications. Various methods confirm ETT position; whilst chest radiography remains standard for identifying ETT depth, ultrasonography (USG) shows promise as a real-time, radiation-free alternative. That is why we aimed to evaluate the concordance between tracheal ultrasound (TUS) and chest x-ray to identify the depth of the ETT in the trachea in preterm neonates and to compare the reproducibility of TUS done by a trainee neonatologist and an expert radiologist. This cross-sectional study involved 85 preterm infants who had an ETT. Tracheal ultrasonography was performed by a neonatologist (who had been trained in chest ultrasonography for 3 months) and repeated by an expert radiologist. Operators were blinded to the radiographic results. The overall agreement was good, with near-complete concordance for ETT localization at the 2nd intercostal space across techniques: 89.4% (PSA 93.5%) between expert TUS and CXR, 82.4% (PSA 89.1%) between trainee TUS and CXR, and 89.1% (PSA 92.3%) between expert and trainee TUS.

*Conclusion*: Bedside tracheal ultrasonography is a reliable, fast, safe, and effective method for assessing endotracheal tube position in the NICU. With adequate training, neonatologists can perform the procedure independently which speeds up ETT position confirmation.

*Trial registration*: The clinical trial was retrospectively registered on clinical trials.gov 7/17/2025. CLINICALTRIALS.GOV ID: NCT07073105. https://CLINICALTRIALS.GOV/STUDY/NCT07073105.

**Table:** 

**What is Known:** • *Improper endotracheal tube (ETT) placement and depth in the trachea can cause severe complications.* • *Chest X-ray is used for the identification of ETT depth in the trachea but causes delays and radiation exposure.*
**What is New:** • *Tracheal ultrasound offers rapid, radiation-free confirmation.* • *With brief training, a neonatology trainee achieved agreement with an expert radiologist in identifying the depth of ETT in the trachea in preterm infants.*

**Supplementary Information:**

The online version contains supplementary material available at 10.1007/s00431-025-06626-3.

## Introduction

Newborns in the NICU (neonatal intensive care unit) are intubated for various reasons, and the majority of these patients undergo chest X-rays (CXRs) several times during their ICU admission to confirm endotracheal tube (ETT) placement, depth in the trachea, monitor the severity of cardiopulmonary illness, and detect complications of other implanted devices [1]. Rapid confirmation of correct tube placement is important because tube malposition is associated with serious adverse outcomes. End-tidal carbon dioxide (EtCO_2_) monitoring is the gold standard for confirming endotracheal tube placement, whereas CXR is used to verify its precise intratracheal position; however, it is often delayed until after ventilation has commenced. Hence, point-of-care methods to confirm correct tube placement have been developed [2].

Although CXRs have limited radiation exposure, frequent CXRs in NICU infants, particularly preterm, result in an accumulated radiation burden over time. This can cause tissue damage and increases the chance of cancer [3]. Tracheal ultrasonography (TUS) is a technique for confirming placement and correct depth of endotracheal intubation. Potential advantages include detection of both main 2 bronchial stem and esophageal intubation, a shorter time to picture availability than CXR, and less radiation [4]. Emergency physicians and anesthesiologists are increasingly using point-of-care ultrasonography (POCUS) on the anterior neck to detect endotracheal and esophageal intubation. It is more accurate and faster than physical examination and capnography, with adult meta-analyses indicating sensitivity of 93–98% and specificity of 97–98% [5]. Several studies are needed to confirm this issue; that is why we aimed to evaluate the concordance between tracheal ultrasound vs. chest x-ray to identify the depth of the ETT in the trachea in preterm neonates, the time required for the communication of TUS and CXR results and also, to compare the reproducibility of tracheal ultrasound done by a trainee neonatologist vs. an expert radiologist.

## Methods

At the Intensive Care Unit for Neonates of Ain Shams University Pediatrics Hospital, Cairo, Egypt, this cross-sectional study was carried out on 85 preterm neonates less than 35 weeks’ gestational age who were about to start invasive mechanical ventilation via ETT. Neonates with upper air way anomalies or whom the caring physician deemed enrollment would disrupt the patient’s care were excluded from the study. The study was started after the Research Ethics Committee’s consent was gained and after obtaining written informed consent from either parents or the legal guardians of the patient.

### Study procedures and study interventions

All patients underwent chest X-ray (CXR) after initial or repeat intubation. The CXR plate was placed before performing the ultrasound, with anterior–posterior radiographs taken in a supine position, ensuring no neck flexion or rotation to avoid changing the ETT position. The site of the ETT tip on CXR was defined according to the corresponding intercostal space for comparison with tracheal ultrasonography. The time for CXR was recorded from giving the request to the time of interpretation by the senior attending neonatologist or radiologist, excluding the time for ultrasound by the expert and trainee. The radiology technician and physician interpreting the CXR were blinded to the ultrasound results.

All infants had TUS performed by an expert radiologist and a trainee neonatologist using a portable ultrasound machine (ACUSON P500), simultaneously with the chest X-ray. The following technique was developed based on prior departmental experience and previously described suprasternal and parasternal ultrasound methods. The method was refined through repeated clinical application to ensure consistent, reproducible imaging quality. A high-frequency linear probe was used for transverse views to evaluate the ETT tip. The training program was conducted by a specialized radiologist and a self-taught neonatologist. The neonatologist had been trained for three months to perform the tracheal ultrasound (TUS). TUS was done after the CXR plate was placed but before the X-ray, with the X-ray taken immediately following the ultrasound to avoid ETT displacement. Pre-warmed gel was applied to the probe, which was placed transversely on the suprasternal notch. The probe was angled slightly lateral or oblique to the right parasternal border to visualize the trachea while minimizing sternal artifacts, to minimize acoustic shadowing. This positioning provided a clear view of the trachea and ETT without significant interference from the sternum or ribs. Minimal probe pressure was maintained to avoid distortion of the imaging field. The esophagus was identified posterior to and to the left of the trachea; this ensured that the ETT was correctly placed in the trachea and confirmed by the absence of a double tract sign [6, 7] (S. Figure 1, 2).

The ETT tip was identified after producing a gentle motion of less than 2 mm, in and out for better visualization of the tip position. This minimal 2 mm ETT adjustment was performed once under direct neonatologist supervision to enhance tip visualization without causing displacement or instability. The tube appeared as a semicircular structure visible within the lumen of the trachea with a posterior shadow that extended down. We moved the probe down until this shadow ended; at this point, the tip of the ETT was identified, and we determined the corresponding intercostal space to be compared to CXR (S Fig. 1–2, S. video 1).

This method proved to be the most suitable, easiest, and fastest for trainees. The time taken by trainees and experts to determine the ETT tip was recorded and compared with CXR times. Operators were not informed of the radiological evaluation outcomes. The time for both investigations was recorded for each patient, and infants with unclear ETT tips on ultrasound were noted but excluded from the sample size. The expert radiologist re-evaluated the ETT position using ultrasound to compare with the trainee’s results. Demographic and physiological data, including age, gender, weight, ETT size, insertion depth, medications during intubation, vital data (recorded non-invasively) at the time of CXR and ultrasound, were documented.

### Statistical analysis

Data were collected, revised, coded and entered into the Statistical Package for Social Science (IBM SPSS 27). Quantitative data were expressed as mean ± SD and range, and qualitative variables as numbers and percentages. Group comparisons for qualitative data used the chi-square or Fisher’s exact test, as appropriate. Paired *t*-tests and repeated-measures ANOVA were applied for quantitative comparisons between two or more paired groups, respectively. Agreement between ultrasound and CXR was assessed using Cohen’s kappa, ICC, and Gwet’s AC1 with 95% confidence intervals. Overall agreement and proportion of specific agreement (PSA) were also calculated, with agreement levels categorized as poor (< 0.20), fair (0.21–0.40), moderate (0.41–0.60), substantial (0.61–0.80), or almost perfect (> 0.80).

## Results

This cross-sectional study was conducted on 85 preterm neonates (150 eligible neonates with 65 excluded as in S. Figure 3). There were 39 females (45.9%) and 46 males (54.1%) with age ranging from 2 to 29 days and with a median (IQR) of 12 (7–16). The most common cause for intubation among the studied patients was respiratory distress syndrome in 55 patients (64.7%) followed by congenital heart disease in 36 patients (42.4%) (Table 1).

**Table 1:** Demographic data and characteristics of the studied patients

		Total no. = 85
**Age (days)**	Median (IQR)	12 (7‒16)
	Range	2‒29
**Sex**	Female	39 (45.9%)
	Male	46 (54.1%)
**Gestational age (weeks)**	Mean ± SD	32.12 ± 1.83
	Range	27‒35
**Weight (kg)**	Mean ± SD	1.61 ± 0.36
	Range	0.9‒2.4
**Sedated or not**	Sedated	85 (100%)
**ETT size (diameter)**	Mean ± SD	3.12 ± 0.38
	Range	2‒3.5
**ETT depth (cm)**	Mean ± SD	8.06 ± 0.64
	Range	7‒9.5
**Reason for intubation**	RDS	55 (64.7%)
	CHDS	36 (42.4%)
	PHTN	6 (7.1%)
	PDA	4 (4.7%)
	HIE	7 (8.2%)
	AOP	6 (7.1%)

*RDS*, respiratory distress syndrome; *CHDS*, congenital heart diseases; *PHTN*, pulmonary hypertension; *PDA*, patent ductus arteriosus; *HIE*, hypoxic ischemic encephalopathy; *AOP*, apnea of prematurity

There was a good overall agreement between the results by the expert TUS and CXR of 89.4%, with kappa agreement of 0.798 (95% CI 0.671 to 0.925) which indicates a very good agreement, PSA was highest for the second intercostal space (ICS) and was 93.5% indicating an almost perfect agreement with CXR, Gwet’s AC1 was 0.811 (95% CI 0.695–0.97), indicating an almost perfect agreement also (Table 2, S Fig. 4).

**Table 2:** Agreement between results of CXR and results of expert and trainee TUS

		CXR				Overall agreement	Kappa (95% CI)	PSA	Gwet’s AC1 (95% CI)
		1st	2nd	3rd	4th				
		No. = 13	No. = 61	No. = 8	No. = 3				
**Result of expert TUS**	**1st**	9 (69.2%)	2 (3.3%)	0 (0.0%)	0 (0.0%)	76 (89.4%)	0.798 (0.671 to 0.925)	75.0%	0.811 (0.695–0.927)
	**2nd**	4 (30.8%)	58 (95.1%)	1 (12.5%)	0 (0.0%)			93.5%	
	**3rd**	0 (0.0%)	1 (1.6%)	7 (87.5%)	1 (33.3%)			82.35%	
	**4th**	0 (0.0%)	0 (0.0%)	0 (0.0%)	2 (66.7%)			80.0%	
**Result of trainee TUS**	**1st**	6 (46.2%)	3 (4.9%)	0 (0.0%)	0 (0.0%)	70 (82.4%)	0.641 (0.468 to 0.814)	54.5%	0.703 (0.566–0.840)
	**2nd**	7 (53.8%)	5 (93.4%)	3 (37.5%)	0 (0.0%)			89.1%	
	**3rd**	0 (0.0%)	1 (1.6%)	5 (62.5%)	1 (33.3%)			66.7%	
	**4th**	0 (0.0%)	0 (0.0%)	0 (0.0%)	2 (66.7%)			80.0%	

*1st*, first intercostal space; *2nd*, second intercostal space; *3rd*, third intercostal space; *4th*, fourth intercostal space; *CXR*, chest xray; *TUS*, tracheal ultrasound

As for the agreement between the trainee neonatologist TUS and the CXR, the overall agreement was good with 82.4%, kappa agreement of 0.641 (95% CI 0.468 to 0.814) which indicates a good agreement, PSA was highest for 2nd ICS and was 89.1% indicating an almost perfect agreement and Gwet’s AC1 0.103 (95% CI 0.566–0.84), indicating a substantial agreement with CXR (Table 2, S Fig. 5).

The agreement between the expert and trainee neonatologist TUS was along the same line; the overall agreement was good with 88.2%, kappa agreement of 0.745 (95% CI 0.588 to 0.903) which indicates a very good agreement, PSA was highest for 2nd ICS with 92.3% indicating a near total agreement and Gwet’s AC1 was 0.806 (95% CI 0.692–0.920), which is just under a near total agreement (Table 3, S Fig. 6).

**Table 3:** Agreement between results of expert and results of trainee by ultrasound

		Results of expert TUS				Overall agreement	Kappa (95% CI)	PSA	Gwet’s AC1 (95% CI)
		1st	2nd	3rd	4th				
		No. = 11	No. = 63	No. = 9	No. = 2				
Result of trainee TUS	1st	7 (63.6%)	2 (3.2%)	0 (0.0%)	0 (0.0%)	75 (88.2%)	0.745 (0.588 to 0.903)	70.0%	0.806 (0.692–0.920)
	2nd	4 (36.4%)	60 (95.2%)	3 (33.3%)	0 (0.0%)			92.3%	
	3rd	0 (0.0%)	1 (1.6%)	6 (66.7%)	0 (0.0%)			75.0%	
	4th	0 (0.0%)	0 (0.0%)	0 (0.0%)	2 (100.0%)			100.0%	

*1st*, first intercostal space; *2nd*, second intercostal space; *3rd*, third intercostal space; *4th*, fourth intercostal space; *TUS*, tracheal ultrasound

Expert TUS was found to be the fastest to be performed compared to both trainee TUS and CXR with 7.91 ± 2.23, 11.02 ± 2.19, and 41.26 ± 8.24 respectively with a *p*-value < 0.001. Also, trainee TUS was significantly faster than CXR with a *p*-value < 0.001 (S Table 1, S Fig. 7).

As regards the vital data of the patients before, during and after the expert and trainee TUS, there was a statistically significant increase in systolic blood pressure (SBP) after ultrasound than during and before with *p*-value < 0.001 and 0.006 respectively for expert TUS and 0.215 and 0.006 respectively for trainee TUS. Also, oxygen saturation (%) was decreased during ultrasound than before with *p*-value 0.001 or less for both trainee and expert. The percentage of patients with mild grimace was increased during TUS [9 (10.6%)] than before and after [0 (0.0%)] with *p*-value < 0.001 for expert and [11 (12.9%)] for trainee TUS. It should be noted that the decrease in oxygen saturation and the change in SBP during TUS was within normal range for GA and age (S Table 2, 3).

## Discussion

Accurate tube placement is vital for the survival of preterm infants in NICUs, particularly during emergencies requiring prompt intervention. While chest X-ray (CXR) can verify the precise intratracheal position of endotracheal tubes, it is often time-consuming and may delay critical, life-saving procedures. Furthermore, repeated use of CXR is commonly required following endotracheal tube changes, clinical deterioration during invasive mechanical ventilation, or placement of devices such as umbilical catheters and central venous lines. This repeated exposure subjects neonates to cumulative doses of ionizing radiation, which may pose long-term health risks [8].

Tracheal ultrasonography (TUS), when performed by an expert radiologist and trainee neonatologist, showed an overall good agreement with CXR with a near total agreement when identifying the tube in the 2nd ICS. Similarly, there was a good agreement between the expert and trainee TUS with a substantial agreement for identifying the ETT in 2nd ICS. These findings support the effectiveness of tracheal ultrasonography—whether performed by an expert or a trainee—in accurately determining ETT depth in preterm neonates.

Many previous studies have debated the utility of ultrasonography (US) in detecting endotracheal tube (ETT) position compared to chest X-ray in newborns. A study by Dennington et al. [9] builds on earlier findings, suggesting that ultrasound can be a reliable tool for assessing ETT placement. They found that bedside ultrasound demonstrated a relatively strong correlation with radiographic findings, with an *r*^2^ value of 0.68.

Similarly, Milling et al. [10] demonstrated the effectiveness of bedside ultrasonography in detecting esophageal intubations. Galiciao et al. [11] reported that the presence of a comet sign or a group of parallel lines on transverse and longitudinal views, respectively, allows for reliable visualization of the ETT in all scenarios. This technique was previously described by Drescher et al. [12]. Slovis et al. [13] were among the first to study ultrasound-based confirmation of ETT position in neonates. They described the location of the tube tip in relation to the aortic arch on the midsagittal plane, utilizing the close anatomical relationship between the carina and the aortic arch.

The presence of a statistically significant reduction in procedure time for both the expert (7.91 ± 2.23 min) and trainee (11.02 ± 2.19 min) compared with CXR (41.26 ± 8.24 min), with *p* < 0.001 for both comparisons, suggests that TUS is a rapid imaging tool capable of delivering timely results, making it advantageous over CXR in critical care settings. Moreover, the relatively short duration of trainee-performed TUS highlights its potential reliability and practicality for use in neonatal intensive care units (NICUs). It should be noted that the time required for obtaining chest X-rays in our unit may have been prolonged, as the procedure is strictly performed by a radiology technician rotating between high-demand areas, whereas the TUS examinations were conducted immediately by the on-site trainee neonatologist; this limitation reflects our local workflow and may not be generalizable to all NICU settings.

In concordance with our results, Najib et al. [8] found that the mean (SD) time gap between intubation and radiographic evaluation was 2 (1) h, but the time necessary for US assessment was less than 5 min.

During the performance of chest X-ray and ultrasound examinations, it is important to monitor patients’ vital signs, including heart rate, blood pressure, and oxygen saturation. This issue was thoroughly addressed in our study, which demonstrated only a minimal increase in systolic blood pressure, slight facial expressions of discomfort, and a mild decrease in oxygen saturation. It is important to note that these changes were merely numerical variations, all remaining within the patients’ normal physiological range, and no complications were observed. To the best of our knowledge, this specific aspect has not been investigated in previous studies, limiting the possibility of direct comparison.

One of the important aspects in our study lies in its focus on preterm neonates, a particularly vulnerable population that is more susceptible to the consequences of delayed or mistimed interventions. In addition, we also monitored the vital signs during tracheal ultrasound and chest X-ray examinations. Additionally, the comparison between trainee- and expert-performed tracheal ultrasound is a novel aspect of this research, which, to the best of our knowledge, has not been previously explored in this specific context.

Before drawing conclusions, several limitations of this study should be acknowledged. One notable limitation is the sample size, as the study was limited to neonates admitted to the neonatal intensive care unit at Ain Shams University Hospitals. Therefore, the generalizability of the findings is restricted, and validation through larger, multicenter studies involving diverse populations is warranted. It should be noted that chest radiography, although widely used, is an imperfect reference method for determining endotracheal tube depth, as beam angulation and patient positioning may lead to misrepresentation of the actual tube level. Therefore, the comparison between TUS and chest radiography in this study reflects concordance rather than diagnostic accuracy against a true gold standard. Additionally, the effectiveness of structured training programs for tracheal ultrasonography was not explored in this study. Future research should investigate the impact of such educational initiatives on the accuracy and reliability of this modality when performed by trainees.

## Conclusion

Tracheal ultrasonography, when compared with standard-of-care methods, possesses many of the key attributes of an ideal modality for identifying endotracheal tube depth in NICUs: it is accessible, rapid, and reliable. It can be easily performed at the bedside and requires only minimal training for neonatologists, making it highly suitable for routine use in critical care settings.

## Supplementary Information

Below is the link to the electronic supplementary material.

Supplementary file1 (DOCX 1507 KB)

## Data Availability

All data generated or analyzed during this study are included in this published article. The data sets generated and analyzed for the study are available from the corresponding author upon reasonable request.

## Abbreviations

AOP: Apnea of prematurity
CHDS: Congenital heart diseases
CXR: Chest X-ray
DBP: Diastolic blood pressure
ET: Endotracheal
ETT: Endotracheal tube
EtCO_2_: End-tidal carbon dioxide
GA: Gestational age
GCS: Glasgow Coma Scale
HR: Heart rate
HS: Highly significant
ICS: Intercostal space
NICU: Neonatal intensive care unit
PDA: Patent ductus arteriosus
PHTN: Pulmonary hypertension
POCUS: Point-of-care ultrasound
RDS: Respiratory distress syndrome
RR: Respiratory rate
SAT: Oxygen saturation
SBP: Systolic blood pressure
TUS: Tracheal ultrasound/ultrasonography
US/USG: Ultrasound/ultrasonography
