# Supplementary material for: Confirmation and reproducibility of endotracheal tube position by tracheal ultrasonography in preterms: expert versus trainee
Source: Eur J Pediatr. 2025 Dec 9;185(1):2. doi: 10.1007/s00431-025-06626-3 (PMC12689820; doi:10.1007/s00431-025-06626-3)
Supplement: Supplementary file 1 — Supplementary file1 (DOCX 1507 KB) [file 431_2025_6626_MOESM1_ESM.docx]

**Confirmation and Reproducibility of Endotracheal Tube Position by Tracheal Ultrasonography in Preterms: Expert versus Trainee**

**Mariam John Amin Ibrahim ^(1)^, Mohamed Nasr Aldein Elbarbary ^(2)^,**

**George Ezzat Elkess Yacoub ^(3)^, Mona Abdo Mostafa Abdo ^(4)^,**

Corresponding author: Mariam John Amin Ibrahim, [mariam.john@med.asu.edu.eg](mailto:mariam.john@med.asu.edu.eg), ORCID number: 0000-0002-4936-8303

**European Journal of Pediatrics**

**Submission ID: 7eb96179-0a09-4944-a7fe-85bd0e6f4915**

**Online supplement**

**Supplementary tables:**

**Supplementary Table (1):** Comparison between time taken by expert, trainee TUS and CXR

|  | | **Expert TUS** | | **Trainee TUS** | **CXR** | | **Test value** | **P-value** | **Sig.** |
| --- | --- | --- | --- | --- | --- | --- | --- | --- | --- |
|  |  |  |  |  |  |  |  |  |  |
| **Time (min)** | **Mean±SD** | 7.91 ± 2.23 | | 11.02 ± 2.19 | 41.26 ± 8.24 | | 1072.254 | <0.001 | HS |
|  | **Range** | 4 ‒ 14 | | 7 ‒ 15 | 30 ‒ 60 | |  |  |  |
| **Post Hoc Analysis** | | | | | | | | | |
| **Expert vs Trainee** | | | **Expert vs CXR** | | | **Trainee vs CXR** | | | |
| <0.001 | | | <0.001 | | | <0.001 | | | |

Repeated Measures ANOVA followed by post hoc analysis using Bonferoni test, chest x ray (CXR), significant (Sig), P <0.001: Highly significant (HS)

**Supplementary Table (2):** Vital data before, during and after expert TUS

|  | | | **Expert TUS** | | | | **Test value** | **P-value** | **Sig.** |
| --- | --- | --- | --- | --- | --- | --- | --- | --- | --- |
|  |  |  | **Vital data before TUS** | **Vital data during TUS** | **Vital data after TUS** | |  |  |  |
| HR | Mean±SD | | 146.14 ± 13.31 | 147.71 ± 10.72 | 147.89 ± 7.71 | | 2.484 | 0.100 | NS |
|  | Range | | 120 ‒ 176 | 126 ‒ 170 | 132 ‒ 167 | |  |  |  |
| RR | Mean±SD | | 47.34 ± 5.23 | 47.51 ± 5.38 | 47.07 ± 5.47 | | 1.305 | 0.271 | NS |
|  | Range | | 35 ‒ 56 | 35 ‒ 56 | 35 ‒ 56 | |  |  |  |
| SBP | Mean±SD | | 75.86 ± 5.44 | 75.86 ± 4.4 | 77.16 ± 4.14 | | 10.257 | <0.001 | HS |
|  | Range | | 65 ‒ 85 | 67 ‒ 86 | 69 ‒ 88 | |  |  |  |
| DBP | Mean±SD | | 50.49 ± 4.21 | 49.99 ± 2.69 | 50.07 ± 3.11 | | 1.433 | 0.242 | NS |
|  | Range | | 43 ‒ 64 | 43 ‒ 60 | 43 ‒ 61 | |  |  |  |
| SAT (%) | Mean±SD | | 92.82 ± 2.19 | 91.95 ± 1.75 | 92.36 ± 1.93 | | 8.596 | 0.001 | HS |
|  | Range | | 90 ‒ 98 | 90 ‒ 96 | 90 ‒ 97 | |  |  |  |
| Grimace | No | | 85 (100%) | 76 (89.4%) | 85 (100%) | | 18.659 | <0.001 | HS |
|  | Mild | | 0 (0.0%) | 9 (10.6%) | 0 (0.0%) | |  |  |  |
| Post Hoc Analysis | | | | | | | | | |
| **Parameters** | | **Before vs during** | | **Before vs After** | | **During vs After** | | | |
| SBP | | 1.000 | | 0.006 | | >0.001 | | | |
| SAT (%) | | > 0.001 | | 0.214 | | 0.184 | | | |

Repeated Measures ANOVA test. **HR**: heart rate**,** **RR**: respiratory rate**,** **SBP**: systolic blood pressure**, DBP:** diastolic blood pressure **SAT**: saturation

**Supplementary Table (3):** Vital data before, during and after trainee TUS

|  | | **Trainee TUS** | | | | **Test value** | **P-value** | **Sig.** |
| --- | --- | --- | --- | --- | --- | --- | --- | --- |
|  |  | **Vital data before TUS** | **Vital data during TUS** | **Vital data after TUS** | |  |  |  |
| HR | Mean±SD | 146.14 ± 13.31 | 147.84 ± 9.68 | 147.89 ± 7.71 | | 2.656 | 0.093 | NS |
|  | Range | 120 ‒ 176 | 130 ‒ 170 | 132 ‒ 167 | |  |  |  |
| RR | Mean±SD | 47.34 ± 5.23 | 47.61 ± 5.59 | 47.07 ± 5.47 | | 1.767 | 0.181 | NS |
|  | Range | 35 ‒ 56 | 35 ‒ 58 | 35 ‒ 56 | |  |  |  |
| SBP | Mean±SD | 75.86 ± 5.44 | 76.67 ± 4.15 | 77.16 ± 4.14 | | 7.564 | 0.002 | HS |
|  | Range | 65 ‒ 85 | 67 ‒ 85 | 69 ‒ 88 | |  |  |  |
| DBP | Mean±SD | 50.49 ± 4.21 | 50.4 ± 2.18 | 50.07 ± 3.11 | | 0.841 | 0.424 | NS |
|  | Range | 43 ‒ 64 | 45 ‒ 60 | 43 ‒ 61 | |  |  |  |
| SAT (%) | Mean±SD | 92.82 ± 2.19 | 92 ± 1.59 | 92.36 ± 1.93 | | 7.316 | 0.002 | HS |
|  | Range | 90 ‒ 98 | 90 ‒ 96 | 90 ‒ 97 | |  |  |  |
| Grimace | No | 85 (100%) | 74 (87.1%) | 85 (100%) | | 22.992 | <0.001 | HS |
|  | Mild | 0 (0.0%) | 11 (12.9%) | 0 (0.0%) | |  |  |  |
| **Post Hoc Analysis** | | | | | | | | |
| **Parameters** | **Before vs during** | | **Before vs After** | | **During vs After** | | | |
| SBP | 0.041 | | 0.006 | | 0.215 | | | |
| SAT (%) | 0.001 | | 0.214 | | 0.125 | | | |

Repeated Measures ANOVA test**,( HR**)heart rate **,** (**RR)**:respiratory rate **,(** **SBP)**: systolic blood pressure**, (DBP):**diastolic blood pressure **( SAT)**: saturation

**Supplementary Figures and video**

**
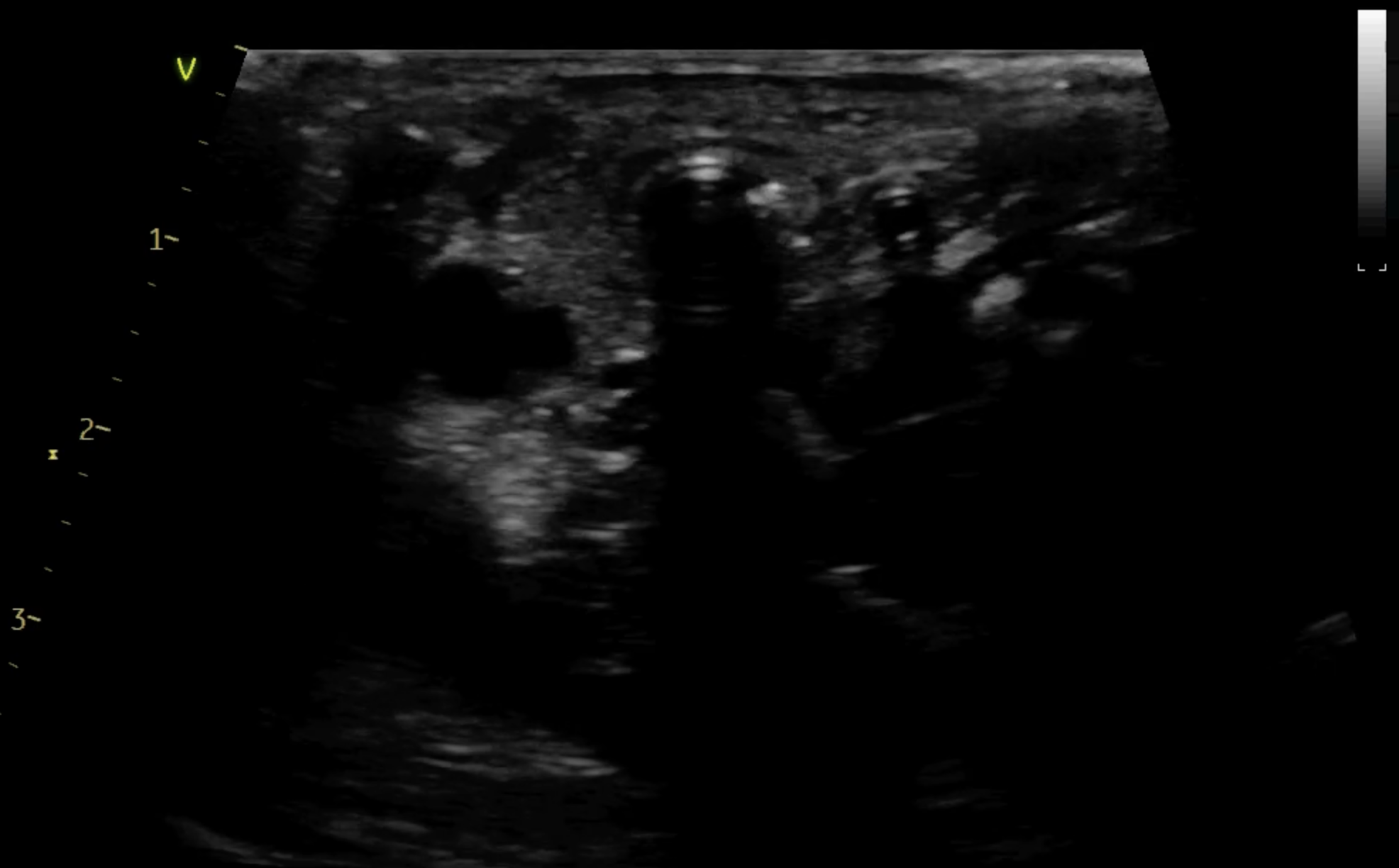
**

**Shadowing**

**Anterior part of ETT causing shadowing**

**Supplementary Figure (1):** Tracheal intubation in ultrasound showing shadowing due to presence of ETT in the trachea


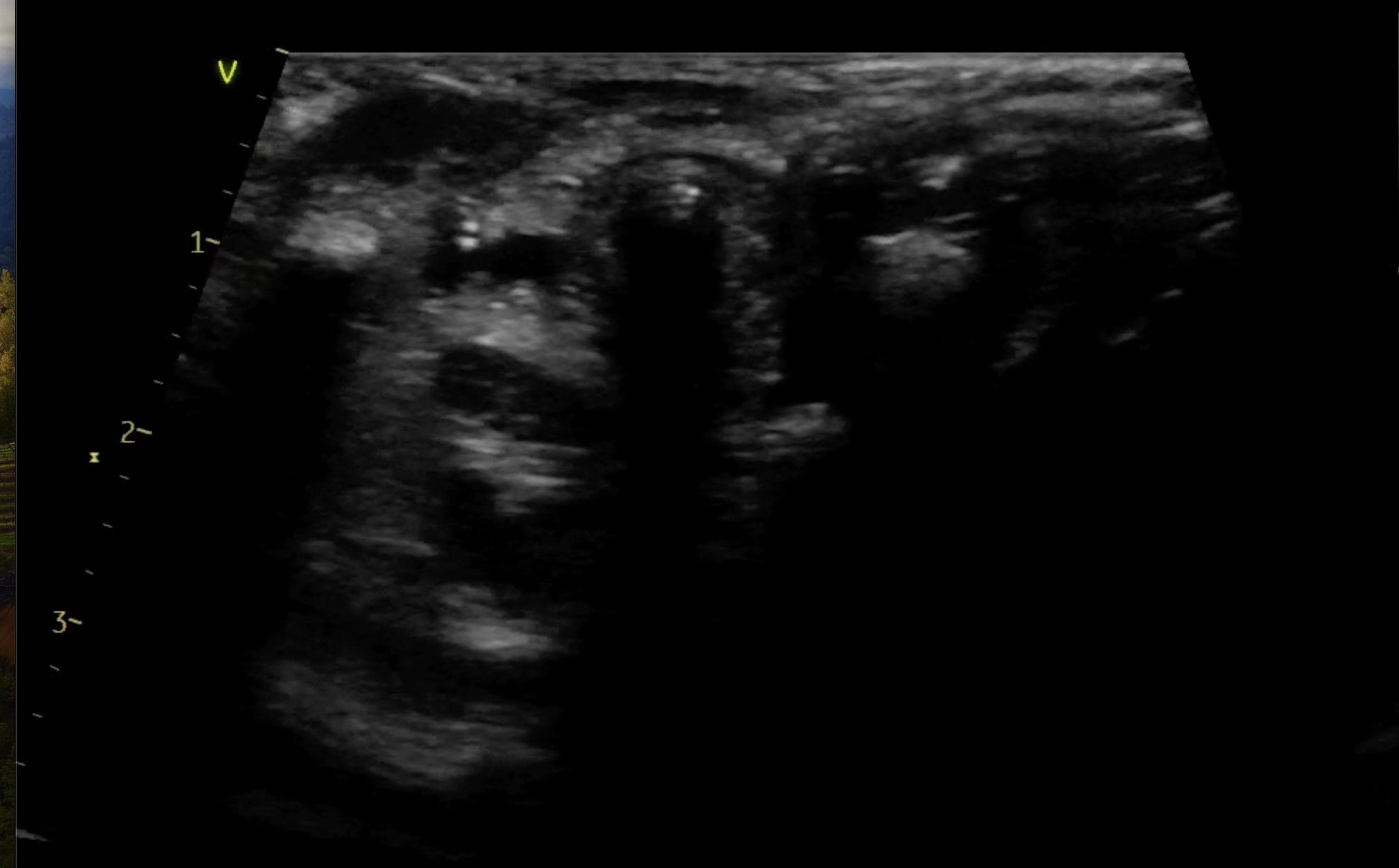


**Trachea without ETT**

**Supplementary Figure (2):** The trachea below the ETT shows absence of shadowing and the appearance of a translucent area. At that level, the tip of the ETT is identified.

**
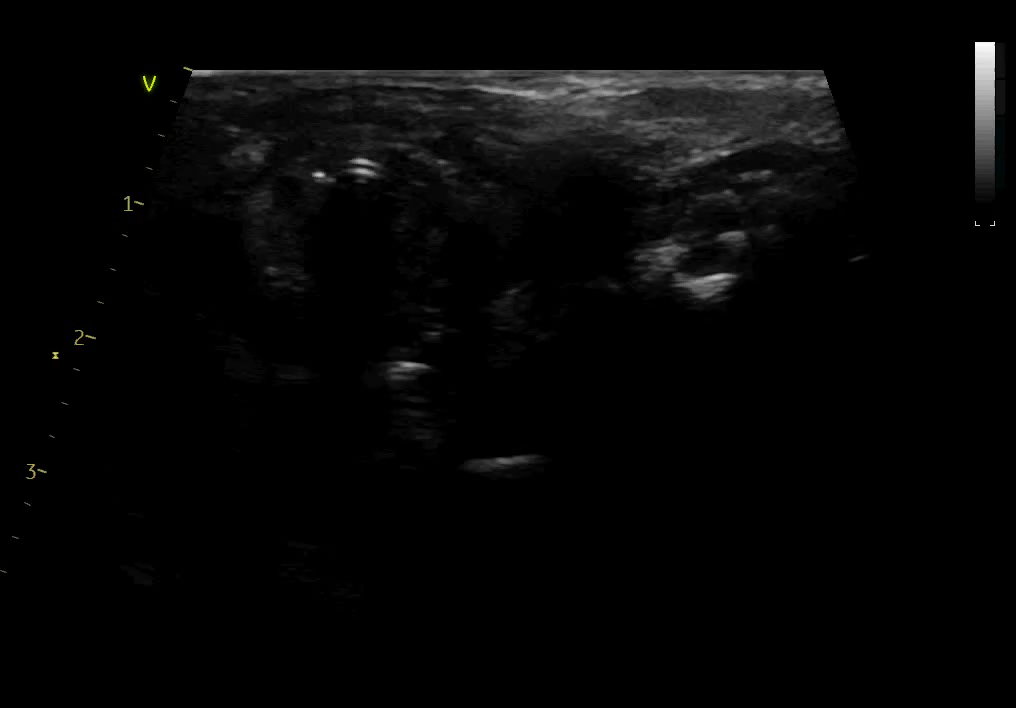
**

**ETT seen inside the trachea and then disapears**

**Supplementary video 1:** This short video demonstrates visualization of the ETT within the trachea. As the probe is moved caudally, the ETT image disappears—indicating that the probe has reached the tip level.

Eligible neonates for this study

(n=150)

Excluded (n=65)

Upper airway anomalies n=7

Decline to participate n=34

Unstable for procedure n=24

Participating neonates (n=85)

Trainee TUS (n=85)

CXR (n=85)

Expert TUS (n=85)

**Supplementary Figure (3): Flow chart of the studied neonates.**

**Supplementary Figure (4) :** Agreement between results of CXR and results of expert TUS

**Supplementary Figure (5):** Agreement between results of CXR and results of trainee TUS

**Supplementary Figure (6):** Agreement between results of expert and results of trainee TUS

**Supplementary Figure (7):** Comparison between time taken by expert, trainee and CXR
